# An Exploratory Prospective Intervention Study on Dietary Content and HbA1c in Experiential Nutrition Guidance for Patients with Type 2 Diabetes

**DOI:** 10.3390/nu18060956

**Published:** 2026-03-18

**Authors:** Katsumi Iizuka, Akemi Ito, Chihiro Ushiroda, Konomi Hirano, Kanako Deguchi, Izumi Hiratsuka, Megumi Shibata, Takeshi Takayanagi, Yusuke Seino, Kokoro Sano, Atsushi Suzuki

**Affiliations:** 1 Department of Clinical Nutrition, School of Medicine, Fujita Health University, Toyoake 470-1192, Japan; chihiro.ushiroda@fujita-hu.ac.jp (C.U.); kanasakuran@gmail.com (K.D.); 2 Food and Nutrition Service Department, Fujita Health University Hospital, Toyoake 470-1192, Japan; akemi305@fujita-hu.ac.jp (A.I.); konomi@fujita-hu.ac.jp (K.H.); 3 Department of Endocrinology, Diabetes and Metabolism, School of Medicine, Fujita Health University, Toyoake 470-1192, Japan; idumi630@fujita-hu.ac.jp (I.H.); megumi03@fujita-hu.ac.jp (M.S.); haratake@fujita-hu.ac.jp (T.T.); seinoy@fujita-hu.ac.jp (Y.S.); aslapin@fujita-hu.ac.jp (A.S.); 4 Shokuno-Okusuri Inc., Tokyo 104-0061, Japan; sano@shokuno-okusuri.com

**Keywords:** teaching kitchen program, Japanese food style, hands-on nutrition education, type 2 diabetes mellitus

## Abstract

**Background:** Medical nutrition therapy (MNT) is crucial for diabetes management. This study evaluated a hands-on nutrition education program that incorporated cooking demonstrations for glycemic control, anthropometrics, and dietary behavior over a 2-month period. **Methods:** Nineteen type 2 diabetes patients (four males and 15 females; 79% female) participated. The median [IQR] age was 70.0 [65.5–73.0] years; the duration of diabetes was 12.0 [8.5–14.0] years; the body mass index (BMI) was 24.3 [21.05–27.90] kg/m^2^, and the baseline HbA1c level was 6.90 [6.55–7.50%]. Approximately 20–25% of patients used injectable therapies. Pre- and post-treatment comparisons were performed via Wilcoxon signed-rank tests, while effect sizes were quantified via rank–biserial correlation (RBC). **Results:** In females (*n* = 15), carbohydrate intake decreased significantly (RBC = −0.583; *p* = 0.050). The protein levels per 1000 kcal exhibited a strong positive effect (RBC = 0.550; *p* = 0.065). HbA1c levels improved significantly (overall: RBC = −0.689 and *p* = 0.009; females: RBC = −0.725 and *p* = 0.014), and while body fat increased significantly (overall: RBC = 0.979 and *p* < 0.001; females: RBC = 0.983 and *p* < 0.001), the skeletal muscle index tended to decrease in females (RBC = −0.333; *p* = 0.268). **Conclusions:** Short-term hands-on nutrition education resulted in significant HbA1c improvement and dietary behavior changes but was accompanied by body composition deterioration. The findings of this study clarify the potential risks of nutritional interventions in elderly diabetic patients and provide important insights for improving future programs.

## 1. Introduction

According to the 2024 National Health and Nutrition Survey, 11 million individuals in Japan are strongly suspected of having diabetes. Among adults aged 20 and older, 12.9% (17.7% of men and 9.3% of women) are affected by the disease [1]. The number of individuals with diabetes has continued to rise: 6.9 million in 1997, 8.9 million in 2007, 9.5 million in 2012, 10 million in 2016, and 11 million in 2024 [1]. While public education and awareness activities related to diabetes have been somewhat effective, the overall incidence trend has continued to increase. According to a 2023 survey, only 5.523 million people are actually receiving treatment [2]. Diabetes is known to cause not only major blood vessel complications, such as cardiovascular and cerebrovascular diseases, but also microvascular complications, such as retinopathy, neuropathy, and nephropathy. Therefore, once someone is diagnosed with diabetes, it is necessary to promptly begin dietary and exercise therapies, as well as treatment with medication.

Dietary therapy is the cornerstone of diabetes treatment [3,4,5,6,7]. Evidence-based dietary approaches, such as carbohydrate counting, the diabetes plate method, and the adoption of healthy dietary patterns (e.g., the Mediterranean diet or the Dietary Approaches to Stop Hypertension [DASH] diet), have been shown to reduce hemoglobin A1c (HbA1c) levels by 0.5% to 2.0% in patients with T2DM [3,4,5]. Registered dietitian nutritionist (RDN)-led medical nutrition therapy (MNT) is considered the gold standard for diabetes nutrition education. Systematic reviews have demonstrated that MNT can lower HbA1c levels by up to 2.0% for 3 to 6 months in patients with T2DM [3,4,5,6,7]. In Japan, the necessary energy intake is established for each age group according to the balance of carbohydrates, fats, and proteins, which differs from overseas guidelines. For this reason, the role of registered dietitians is important.

In contrast, sustaining the effects of nutritional guidance over time is quite difficult [8]. In everyday clinical practice, many patients who receive nutritional guidance often refuse subsequent sessions. Traditional nutritional counseling faces significant challenges in terms of feasibility and implementation. The referral rate from medical institutions to RDNs remains at only 20–50%, and many studies have reported even lower rates ranging from 7 to 37% [8,9,10,11]. The dropout rate among referred patients may be as high as 79%, which highlights the difficulty of maintaining continued attendance at MNT programs [12]. Recently, in addition to classroom-based nutritional guidance, teaching kitchen (TK) programs that incorporate cooking practices have been developed [13,14,15,16]. The TK model was pioneered at the Tulane University School of Medicine, where the Goldring Center for Culinary Medicine established the world’s first medical school-based research-oriented teaching kitchen [13]. Since then, various facilities have conducted clinical trials, which have led to improvements in dietary habits. In Japan, the Teaching Kitchen for Diabetes in Japan (TKD-J) trial is currently underway, but the results have not yet been published [15]. Despite promising findings, a critical gap remains in the literature: most TK studies have focused primarily on glycemic control, dietary behaviors, and quality-of-life outcomes, with limited attention dedicated to body composition changes. Furthermore, we analyzed the dietary habits of healthy individuals according to age group and sex, created menus according to these findings, and provided nutritional guidance to staff and prediabetic patients at the university (Toyoake, Haneda). Through these activities, we found that rather than presenting fixed menus, a focus on side dishes instead of main courses offers a broader range of applications when daily meal planning is considered.

In addition, side dishes play an important role in the intake of dietary fiber within meal plans [17,18,19,20]. Since dietary fiber helps stabilize blood sugar levels, it is believed to aid in the prevention and improvement of diabetes [17]. Side dishes can actively incorporate vegetables, mushrooms, seaweed, and other foods rich in dietary fiber, thereby enhancing the variety of meals and contributing to better blood sugar control [18,19,20]. Therefore, increasing the repertoire of side dishes may lead to greater meal diversity and contribute to improved blood sugar levels.

Therefore, for patients attending the outpatient endocrinology and diabetes clinic at Fujita Health University, in addition to conventional nutritional guidance, we provided cooking classes for side dishes (recipes for side dish preparation). We then explored changes in dietary energy intake, nutrient levels, body composition (body mass index (BMI), skeletal muscle index (SMI), and body fat percentage), HbA1c levels, and serum lipid levels before and after the intervention. This study provides important insights into how experiential nutritional guidance can impact not only glycemic control but also body composition and changes in eating behavior.

## 2. Materials and Methods

### 2.1. Subjects

Twenty-one patients (4 men and 17 women) aged 20 to 74 years with type 2 diabetes who were admitted to the outpatient Endocrinology, Metabolism, and Diabetes Clinic at Fujita Health University Hospital were initially included. Among these 21 patients, 2 were excluded from the study because they were receiving pacemaker treatment and the interval between their blood tests was more than six months. Nineteen patients (4 men and 15 women) aged 20 to 74 years with type 2 diabetes were ultimately included in the study.

The inclusion criteria were as follows: (1) patients who were provided with a sufficient explanation of the study, fully understood the study, and gave written informed consent of their own free will to participate; (2) patients aged 20 to 74 years with type 2 diabetes at the time of consent; and (3) those whose treatment had not changed from the first to the second nutrition counseling session. The exclusion criteria were as follows: (1) patients with severe diabetic complications (such as renal failure or severe proliferative retinopathy), (2) those with dementia, and (3) any other patients deemed inappropriate for the study by the principal investigator.

As for the definition of terms, under the “Act on Securing Medical Care for the Elderly” (Act No. 80 of 1982), individuals aged 65 and over are considered elderly, with those aged 65 to 74 defined as “early-stage elderly” and those aged 75 and over defined as “late-stage elderly” [21].

This study was conducted in accordance with the principles of the Declaration of Helsinki and was approved by the Research Ethics Committee of Fujita Health University (application number: HM23-469; approval date: 4 April 2024). This study was registered with the UMIN Clinical Trial Registry (UMIN000054477, registered on 24 May 2024).

### 2.2. Study Procedure

**Figure 1:**
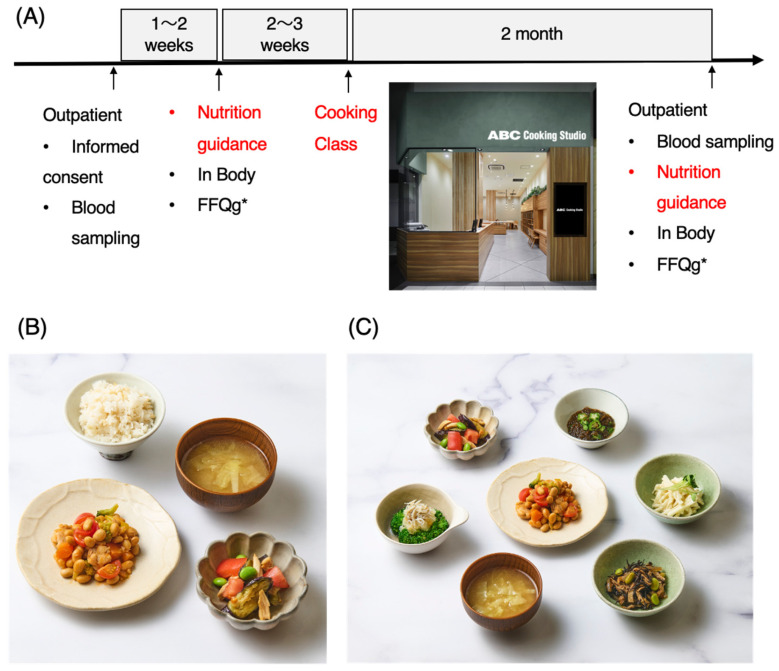
An example of the implementation process of this study and a dish used in the cooking class. (**A**) The process of this study began with obtaining patient consent at the outpatient clinic and drawing blood samples, followed by scheduling nutrition guidance two weeks later. During the nutrition guidance session, a body composition evaluation was conducted using InBody, and a dietary survey was carried out using the FFQg. Two to three weeks after the nutrition guidance, a cooking class was held for practical cooking instruction. At the same time, small side dishes were also taught. Finally, blood was drawn again, and during the nutrition guidance session, body measurements and a dietary survey using the FFQg were conducted once more. (**B**) Dish tasted during the cooking class (chicken and beans, miso soup, steamed chicken and tomato salad, and multigrain rice). (**C**) Example of a side dish: ① chicken and beans, ② eggplant salad, ③ broccoli with grated daikon, ④ okra and mozuku seaweed in vinegar, ⑤ daikon with plum sauce, and ⑥ simmered hijiki seaweed. * FFQg: Food Frequency Questionnaire based on food groups.

Blood samples and consent forms were obtained during the outpatient visit. One week later, during the first nutrition counseling session, body composition was measured with InBody 770 (Seoul, Republic of Korea) (including weight, BMI, skeletal muscle mass, and body fat percentage), and a food intake frequency survey was conducted via the FFQg [22]. Furthermore, two weeks after nutritional counseling, cooking instructions were provided. Two months after the initial blood draw, a second nutritional counseling session was held, after which another FFQg food intake frequency survey was given, followed by blood sampling and body composition measurement (Figure 1).

### 2.3. Blood Collection

HbA1c and serum lipid levels were measured during outpatient visits and were collected from patients in a nonfasting state. The data obtained were age (years); body mass index (BMI, kg/m^2^); and total cholesterol (TC, mg/dL), high-density lipoprotein cholesterol (HDL-C, mg/dL), triglyceride (TG, mg/dL), non-HDL-C (mg/dL), and HbA1c concentrations (%). The height and body mass of the participants were measured by a nurse during the examinations, and the lipid and HbA1c levels were measured in a laboratory at the same hospital. The plasma lipid concentrations (TC, TG, and HDL-C) were measured using a Hitachi LABOSPECT008 (Hitachi High-Tech Corporation, Tokyo, Japan), and HbA1c levels were measured using an A1c HA-8190 (Arkray, Kyoto, Japan). Non-HDL-C concentrations were calculated from the TC and HDL-C concentrations.

### 2.4. Food Frequency Questionnaire Based on Food Groups (FFQg)

The FFQg (Food Frequency Questionnaire based on food groups) is a survey method in the form of a questionnaire designed to estimate the amount and frequency of intake for 29 food groups and 10 types of cooking methods over the past one to two months, with a focus on “habitual dietary intake” [22]. This questionnaire has also been used in many studies involving Japanese participants [23,24,25,26,27,28,29].

### 2.5. Nutritional Guidance

Nutritional guidance sessions comprised a 30 min one-on-one diabetes nutrition counseling session conducted by a registered dietitian, with a total of five dietitians involved. The session included an assessment of dietary intake, an explanation of body composition measurement results, dietary guidance specifically for diabetes, appropriate energy intake guidance (including guidance on rice portion sizes and protein sources), and advice on snacks. The average recommended energy intake was set at 25–30 kcal per day per ideal body weight (IBW). Three first-time participants and 16 repeat participants were involved, for a total of 19 individuals.

### 2.6. Cooking Class

In the cooking class held at ABC Cooking Studio, under the guidance of the cooking instructor, participants prepared and sampled chicken and beans, miso soup, steamed chicken and tomato salad, and multigrain rice within 45 min. The energy and nutrients contained within the sampled dishes were 400 kcal, 19.3 g of protein, 6.9 g of fat, 68.7 g of carbohydrates, 8.2 g of dietary fiber, and 1.9 g of salt equivalent. In addition, recipes for side dishes to be made at home were distributed. These menu items are served at Fujita Health University Hospital and include the following: ① chicken and beans (109 kcal, protein: 10.8 g, fat: 4.3 g, carbohydrate: 10.0 g, fiber: 3.6 g, and NaCl: 0.8 g), ② eggplant salad (46 kcal, protein: 3.6 g, fat: 1.5 g, and NaCl: 0.5 g), ③ broccoli with grated daikon (27 kcal, protein: 3.1 g, fat: 0.3 g, carbohydrate: 5.1 g, fiber: 2.6 g, and NaCl: 0.4 g), ④ okra and mozuku seaweed in vinegar (19 kcal, protein: 0.3 g, fat: 0.1 g, carbohydrate: 4.5 g, fiber: 1.1 g, and NaCL: 0.3 g), ⑤ daikon with plum sauce (47 kcal, protein: 0.5 g, fat: 3.1 g, carbohydrate: 5.1 g, fiber: 0.9 g, and NaCl: 0.3 g), and ⑥ simmered hijiki seaweed (52 kcal, protein: 2.4 g, fat: 3.2 g, carbohydrate: 4.7 g, fiber: 2.0 g, and NaCl: 0.7 g).

### 2.7. Statistical Analysis

Variable classification and study population

We compared preintervention (1st) and postintervention (2nd) measurements for the 19 participants (overall cohort) and for the 15 female participants (Sex = 1). The analyzed variables were classified into three categories:

Anthropometric measures: Body weight (BW), body mass index (BMI), body fat percentage (BF), and skeletal muscle index (SMI).

Blood parameters: Glycated hemoglobin (HbA1c), triglyceride (TG), high-density lipoprotein cholesterol (HDL-C), and low-density lipoprotein cholesterol (LDL-C) levels.

Nutritional intake: Energy, protein, fat, carbohydrates, and dietary fiber.

### 2.8. Statistical Methods

Pre- and postintervention comparisons were performed via the paired Wilcoxon signed-rank test. Given the small sample size and potential violation of normality assumptions, we selected a nonparametric approach. Effect sizes were quantified via rank–biserial correlation (RBC) [30,31,32]. The RBC ranges from −1 to +1, where larger absolute values indicate larger effect sizes. The conventional interpretation thresholds are as follows: |RBC| < 0.3 (small effect), 0.3 ≤ |RBC| < 0.5 (moderate effect), and |RBC| ≥ 0.5 (large effect) [32].

### 2.9. Descriptive Statistics and Handling of Missing Data

Continuous variables are presented as medians [interquartile ranges (IQRs)]. Only complete pairs with measurements at both time points were included in the analysis. The number of pairs with zero differences (ties) was recorded, and the effective sample size (n_eff_nonzero) was reported. Variables with fewer than three effective pairs were excluded from statistical tests.

### 2.10. Statistical Software

All the statistical analyses were conducted via R version [R version 4.5.2] (R Foundation for Statistical Computing, Vienna, Austria) [33]. Data manipulation utilized the dplyr, tidyr, and readr packages, and the ggplot2 package was used for visualization. The Wilcoxon signed-rank test was implemented via the wilcox.test() function with paired = TRUE and exact = FALSE arguments.

Given the small sample size (n = 19) and exploratory nature of this study, we did not apply multiple testing corrections (e.g., false discovery rate adjustment). Instead, we reported effect sizes (rank–biserial correlation, RBC) alongside *p*-values to provide a comprehensive interpretation. Statistical significance was set at *p* < 0.05.

## 3. Results

### 3.1. Background of the Participants

Nineteen participants were involved in this study (four men and 15 women). The median [interquartile range] age was 70.00 [65.50, 73.00] years; the duration of diabetes was 12.00 [8.50, 14.00] years, and the BMI was 24.30 [21.05, 27.90] kg/m^2^, which is relatively high. The median [interquartile range] HbA1c level was 6.90 [6.55, 7.50]% (Table 1). Among the participants, 26% (five individuals) used insulin, and 21% (four individuals) used GLP-1 receptor agonists. In summary, the participants were predominantly female (79%) and had been living with diabetes for approximately 10 years, and approximately 20–25% were receiving treatment that consisted of injectable medications. Therefore, in the following sections, data will be presented for both the entire group and for women only.

### 3.2. Before-And-After Comparisons of Energy and Nutrient Intake

The FFQ was evaluated using the Wilcoxon signed-rank test. Pre- and postintervention comparisons of dietary energy and nutrient intake were conducted, and the effect size was calculated via RBC. In the overall group (n = 19), only differences in carbohydrate intake had a moderate effect size (RBC = −0.379; *p* = 0.153) (Table 2). A statistically significant decrease in carbohydrate content was observed (RBC = −0.583; *p* = 0.050) in the female group (n = 15), which indicates a large effect size. No statistically significant differences were found for energy (RBC = −0.400; *p* = 0.182), fat (RBC = −0.400; *p* = 0.182), or dietary fiber (RBC = −0.367; *p* = 0.222), although moderate effect sizes were observed. In contrast, differences in protein intake had a small effect size (RBC = −0.133; *p* = 0.670) (Table 2). All the values were negative, indicating a tendency for intake to decrease after the intervention. Next, to eliminate the influence of total energy intake, nutrient intake per 1000 kcal was compared pre- and postintervention. In the female-only group, while protein per 1000 kcal did not significantly differ, a large positive effect size was observed (RBC = 0.550; *p* = 0.065) (Table 3). Therefore, since the intake of nutrients other than protein decreased after the intervention, the relative proportion of protein consumed tended to increase.

### 3.3. Before-And-After Comparison of Body Composition Indicators

A pre- and postintervention comparison of body composition indicators (body fat percentage, BMI, BW, and SMI) was conducted via the Wilcoxon signed-rank test, and the effect size was calculated via RBC. In the overall group (n = 19), differences in body fat percentage had a very large positive effect size that demonstrated a statistically significant increase (RBC = 0.979; *p* < 0.001). For BMI (RBC = 0.419; *p* = 0.176) and BW (RBC = 0.412; *p* = 0.155), although the differences were not statistically significant, medium effect sizes were observed (Table 4). In the female group (n = 15), differences in body fat percentage again showed a very large positive effect size, with a statistically significant increase (RBC = 0.983; *p* < 0.001). With respect to the SMI, while no statistically significant difference was found, a medium negative effect size was observed (RBC = −0.333; *p* = 0.268), which suggests a decreasing trend (Table 4). Therefore, the body fat percentage increased significantly regardless of sex, while the SMI tended to decrease among women.

### 3.4. Before-And-After Comparison of Blood HbA1c and Lipid Levels

In the pre and post hoc comparisons of blood test data, HbA1c levels in the overall group (n = 19) showed a large negative effect size, with a statistically significant decrease observed (RBC = −0.689; *p* = 0.009) (Table 5). TG levels also exhibited a moderate negative effect size, but the difference was not statistically significant (RBC = −0.322; *p* = 0.239). In the female group (n = 15), the effect size of HbA1c was strongly negative, and a statistically significant decrease was observed (RBC = −0.725; *p* = 0.014). Therefore, the HbA1c level was significantly improved by the intervention.

## 4. Discussion

In this study, we evaluated the effects of a nutritional and exercise intervention program on 19 patients with type 2 diabetes (79% female, median {IQR} of ages: 70.00 [65.50, 73.00]. A major feature of our intervention is that we taught how to prepare side dishes in combination with nutrition guidance. Many side dishes contain foods rich in dietary fiber, and we considered that the improvement in blood glucose could be achieved through the effects of dietary fiber, which is a novel approach compared to previous studies. With respect to nutritional intake, the female group presented a statistically significant decrease in carbohydrate intake and a decreases in energy, fat, and dietary fiber. Regardless of sex, body fat percentage significantly increased, and the female group tended to have a decreased SMI. Finally, the HbA1c level improved significantly both overall and in the female group. Although the ratio of the relative protein level to the total energy intake, which is an important measurement that indicates improved glycemic control from the intervention, tended to increase (RBC = 0.550; *p* = 0.065), this increase may not have been sufficient to maintain skeletal muscle mass. Therefore, while this intervention improved blood glucose levels, this finding is the first to suggest that the same intervention may worsen body composition, particularly in older women. In the future, intervention strategies that balance blood glucose control with the maintenance of body composition while being tailored to the characteristics of each patient will be necessary.

As the meal composition changed, energy and fat intake decreased, at which point energy was predominantly derived from carbohydrates. As a result, a relative increase in the consumption of protein was observed. In our cooking class, the side dish recipes provided mainly included those for vegetables, seaweed, and soybeans; thus, protein intake did not increase significantly. It is generally known that increased dietary fiber intake can lead to decreased energy intake, but in this study, we did not observe an increase in dietary fiber intake. In Cooking Matters (2023), cooking classes were held weekly for 6 weeks, with a three-month follow-up [14]. The authors reported changes in dietary behavior and reductions in energy and nutrient intake ((energy intake: −348 kcal/day (*p* = 0.013), carbohydrates: −51 g/day (*p* = 0.005), added sugars: −17 g/day (*p* = 0.025), and refined grains: −0.61 oz/day (*p* = 0.041)), which supports our findings. In Cooking Well with Diabetes [16], among patients with type 2 diabetes, after a 4-week cooking class program, the percentage of participants who consumed vegetables at least twice a day increased from 47.0% to 62.2% (*p* < 0.001), and the percentage of those who consumed fruit at least twice a day increased from 34.8% to 49.0% (*p* < 0.001). Moreover, the percentage of those who did not regularly consume soda increased from 47.8% to 50.7% (*p* < 0.001), and the percentage of those who did not consume sugary beverages increased from 41.6% to 47.0% (*p* < 0.001) [16]. These results only partially agreed with our results (2024). Overall, positive outcomes such as reduced carbohydrate intake, decreased energy consumption (a reduction of 300–400 kcal/day), a decrease in added sugars and refined grains, and a relative increase in protein consumption were observed. While the effect of cooking classes alone is unclear, participation in both nutrition guidance and cooking classes likely prompted participants to consider blood glucose management, which indirectly led to changes in their dietary habits. The specific decrease in carbohydrates may reflect the characteristics of diabetes patients who are conscious of their blood sugar (carbohydrate restriction). Given that many of the recipes introduced in the cooking class focused on vegetables, it is possible that the relative intake of staple foods, which are the main source of carbohydrates, decreased. Additionally, since the participants in this study experienced an average disease duration of approximately 12 years and already possessed knowledge regarding dietary therapy, it is possible that the intervention served as an opportunity to put their existing knowledge into practice. Actually, 86% of the participants had already received regular nutritional guidance. The finding that protein intake did not change reflects the effects of nutritional guidance, which were in line with established guidelines. The protein intake per body weight was approximately 1.2 g/BW. Given that the SMI showed a decreasing trend among women, it is important to emphasize the need to ensure adequate absolute protein intake for elderly patients with diabetes. Finally, since guidance was only provided in a single instance, the appropriate frequency and duration of continuous nutritional guidance and cooking classes remain to be determined in future studies.

Our research revealed a significant increase in body fat percentage. According to a review of previous research, most major studies on teaching kitchen programs did not consider weight or body composition as primary endpoints. Programs conducted at Tulane University [13], Cooking Matters for Diabetes [14], and Cooking Well with Diabetes [16] all focused on HbA1c levels and behavioral changes and did not assess changes in body composition. The only exception is the ongoing TKD-J trial [15], which has set body composition as a secondary endpoint, but its results have not yet been published. Therefore, this study aimed to conduct a detailed evaluation of changes in body composition after a cooking class program, which provided important insight into the concomitant improvement in HbA1c levels and the tendency toward increased body fat and decreased muscle mass. Several possible factors underlie the increase in body fat percentage observed in this study. First, 26% (five participants) of the study subjects used insulin, and 21% (five participants) used GLP-1 receptor agonists. Insulin is an anabolic hormone that promotes weight gain and fat accumulation as blood glucose control improves [34]. Actually, a trade-off between improved glycemic control and weight gain has been reported in patients with type 2 diabetes who receive insulin therapy [34,35]. Different diabetes medications can have different effects, as some drugs, such as GLP-1 receptor agonists and SGLT2 inhibitors, improve blood sugar levels while lowering body weight, whereas others, such as insulin, result in initial weight gain that is correlated with improved blood sugar; however, the two become independent later on [36,37,38,39]. Therefore, when interpreting results in patients using various medications, caution is advised. Future studies should conduct subgroup analyses according to the type of pharmacological therapy used in order to evaluate the influence of medications. The participants in this study were elderly, with a median age of 70 years, and 79% were women. Older women are more likely to accumulate visceral fat because of postmenopausal hormonal changes, and the decline in basal metabolism with aging means that body fat tends to increase even with the same caloric intake. Cooking class programs alone may not lead to a substantial increase in physical activity. Whether the increase in body fat percentage and the decrease in SMI observed in this study are transient remains to be clarified, but the cooccurrence of increased body fat percentage and decreased SMI is an important finding that suggests that the risk of sarcopenic obesity and attempts to promote increased physical activity may need to be combined.

Another outcome of this study was the significant improvement in the HbA1c level. This can be attributed to improved blood glucose control resulting from reduced carbohydrate intake. Since the program provided was not the same as previous cooking class programs, a direct comparison is not possible. With respect to short-term effects, Cooking Matters (2023) conducted weekly cooking classes for six weeks with diabetic participants (average age 57; 65% female), followed by a three-month follow-up. No statistically significant differences were observed in the HbA1c levels between the intervention and control groups (immediately after: +0.17% [*p* = 0.501]; three months later: −0.05% [*p* = 0.84]) [14]. However, reductions were observed in dietary behavior and intake: energy intake decreased by −348 kcal/day (*p* = 0.013); carbohydrate intake decreased by −51 g/day (*p* = 0.005); added sugar intake decreased by −17 g/day (*p* = 0.025), and refined grain intake decreased by −0.61 oz/day (*p* = 0.041) [14]. With respect to medium-term effects, Tulane University’s group (Monlezun et al., 2015) implemented Mediterranean diet-based cooking and nutrition education for six months in diabetic patients (average age of 62 years; 67% female) in a teaching kitchen, which resulted in an HbA1c reduction of −0.4% (control group: −0.3%; *p* = 0.57), but the difference before and after the intervention was not statistically significant [13]. Although no studies have been conducted in Japan thus far, research that aims to assess long-term effects is currently underway [15]. In that ongoing study, overweight patients with type 2 diabetes (aged 20–79 years, HbA1c: 6.5–9.0%, BMI ≥ 23 kg/m^2^ or abdominal obesity) participated in a main program (4 months, weekly 2 h sessions for 16 weeks (six in person and 10 online)), a maintenance program (8 months, monthly 2 h sessions for 8 months (four in person, four online)), and follow-up (4 months) based on the observation period, although the results have not yet been reported. Additionally, while the influence of the seasons may be a factor, since the study was conducted in the summer, when changes in HbA1c levels are minimal, the impact of seasonal variation appears to be limited [40].

In summary, the degree of HbA1c improvement (RBC: −0.689; female RBC: −0.725), with a consistent trend toward reduced carbohydrate intake, falls within the range reported in previous studies (−0.2–0.6%). The patient groups in each study are different, and thus direct comparisons are impossible, but an average improvement of approximately 0.2–0.3% can be expected. A 1% reduction in HbA1c levels has been shown to lower the risk of microvascular complications by approximately 37% and the risk of myocardial infarction by approximately 14%; therefore, the degree of improvement observed in this study can be considered clinically meaningful. In addition to simply addressing the problem of excess HbA1c, improving dietary balance and content is important. The reduction in HbA1c levels may reflect not only decreased carbohydrate intake but may also facilitate behavioral changes. In the Cooking Matters for Diabetes program, the intervention group showed significant improvements in diabetes self-care scores and self-efficacy, which suggests greater confidence in the ability of patients to translate knowledge into action [14]. The combination of cooking classes may have provided an opportunity to put nutrition knowledge—previously learned but only theoretical—into practice.

This study has several limitations. First, with 19 participants, the sample size was small, and of these, only four were male (21%), and thus it is not possible to thoroughly assess sex-related differences. Since this was an exploratory study, we did not conduct power analysis. Also, the participants in this study were predominantly women, which is similar to other teaching kitchen programs. This is likely because those involved in cooking are more interested in and protective of such initiatives [41]. Moreover, as a single-institution study, potential sampling bias cannot be excluded [42,43,44,45].

However, this study employed a single-arm design for an exploratory feasibility study in preparation for a future parallel-group randomized controlled trial (RCT) [42,43,44,45]. The primary objectives were (1) to evaluate the feasibility and acceptability of the intervention, (2) to estimate effect sizes for appropriate sample size calculations in a future trial, and (3) to refine the intervention protocol. This approach is internationally recognized for exploratory research [43,44,45], and the CONSORT 2010 extension for pilot and feasibility trials does not mandate statistical power calculations or the inclusion of control groups at this stage [43].

A crossover design was deemed inappropriate for this cooking-based educational intervention because of irreversible learning effects and carry-over bias [46,47,48]. Therefore, our future RCT will adopt a parallel-group design with a waiting-list control or usual care group.

Given the small sample size, we did not apply multiple testing corrections and instead emphasized effect sizes (rank–biserial correlations, RBCs), as recommended for exploratory studies [49]. Although some outcomes (e.g., SMI reduction and RBC = −0.333) showed moderate-to-large effects without statistical significance, these findings warrant validation in a larger-scale trial.

Second, while more pronounced changes were observed in women, suggesting the possible involvement of postmenopausal hormonal changes and metabolic characteristics, our study design did not allow us to explicitly investigate these factors. Third, as subgroup analyses by type of pharmacological treatment (especially insulin and GLP-1 receptor agonist users vs. nonusers) were not conducted, we were unable to evaluate the impact of diabetes medications on changes in body composition. Fourth, although dietary intake was assessed via the Food Frequency Questionnaire based on food groups (FFQg), FFQs are prone to underestimations of actual intake. Additionally, since physical activity was not evaluated, changes in behaviors other than diet before and after the intervention were not examined. We also did not assess glycemic variability via CGM, as is currently being performed in other studies. For example, the Teaching Kitchen for Diabetes in Japan (TKD-J) trial plans to incorporate CGM, physical activity monitoring by Fitbit, and individualized support via a web application [15]. Whether digital health tools can prevent the body composition deterioration observed in our study is unknown, and the long-term sustainability of these tools is also unclear; thus, the current evidence that supports the effectiveness of these tools remains limited. Furthermore, it is unclear whether digital health tools such as CGM and Fitbit are feasible and cost-effective for all diabetes patients. It is important to use simple, economical, and minimally burdensome methods such as daily step counts and diet-recording apps to assess diet and physical activity, and we plan to explore these methods in future research.

In future studies, it will be necessary to conduct studies with larger sample sizes, perform detailed analyses by sex, conduct subgroup analyses according to different pharmacological treatments, maintain detailed records of the content and implementation of exercise interventions, and utilize more accurate dietary assessment methods. In this study, the median age is 65 years, and we plan to conduct an advanced analysis focusing on women aged 60 to 75. Furthermore, it is important to extend the intervention period (e.g., to 6 months or 12 months) to evaluate the long-term sustainability effects and the temporal patterns of changes in body composition. Additionally, the inclusion of supplementary evaluation items such as physical function, inflammatory markers, and indicators of insulin resistance may allow for a deeper understanding of whether changes in body composition indeed represent a pathological issue.

## 5. Conclusions

This study demonstrated that a nutrition and exercise intervention program focused on cooking classes significantly improved HbA1c levels in elderly patients with type 2 diabetes, which indicates a trend toward significant increases in body fat percentage and a tendency toward decreased SMI values. This cooccurrence of “improvement in blood glucose control and worsening of body composition” has not been previously reported in studies on cooking class programs, and thus this is an important finding, particularly as it prompts a re-evaluation of intervention strategies in populations including elderly women and insulin users. Future diabetes education programs should be designed to achieve comprehensive health outcomes, not only to improve glycemic control but also to maintain muscle mass and preserve physical function. Specifically, in addition to assessments of physical activity levels, the combination of effective exercise programs, such as resistance training, may be necessary. Furthermore, the use of not only food frequency questionnaires but also dietary record apps to evaluate dietary intake could allow for a more detailed assessment of dietary patterns. In diabetes research, a reduction in HbA1c levels tends to be the primary outcome measure, but the evaluation of other outcomes such as body composition and dietary balance is also important. Finally, individualized studies that consider factors such as medication treatment, disease duration, and endogenous insulin secretion are needed. The findings of this study reveal the potential risks of nutritional interventions in elderly patients with diabetes and provide important suggestions for the future improvement of such programs.

## Figures and Tables

**Table 1:** Background of the participants in this study.

Variable	Median (IQR) *
Number	19
Sex (female, Sex = 1)	15/19 (78.9%)
Duration of DM	12.00 [8.50, 14.00] *
Age	70.00 [65.50, 73.00] *
Height	155.60 [153.00, 158.75] *
BW_1st	59.90 [47.40, 70.95] *
BMI_1st	24.30 [21.05, 27.90] *
BF_1st	16.70 [11.65, 25.35] *
SMI_1st	6.22 [5.88, 7.19] *
HbA1c_1st	6.90 [6.55, 7.50] *
TG_1st	134.00 [102.50, 158.00] *
HDLC_1st	55.00 [44.50, 63.00] *
LDLC_1st	106.00 [90.50, 123.50] *
Use of anti-diabetes drugs (n(%))	
Insulin analogs	5 (26%)
GLP-1 receptor agonists	4 (21%)
DPPIV inhibitors	8 (42%)
Metformin	9 (47%)
SGLT2 inhibitors	8 (42%)

* Data represented as the median [IQR]. Abbreviation: BW, body weight; BMI, body mass index; BF, body fat percentage; SMI, skeletal muscle mass index; TG, triglyceride; HDLC, high-density lipoprotein cholesterol; LDLC, low-density lipoprotein cholesterol; DPPIV, dipeptidyl peptidase; SGLT2, sodium glucose cotransporter 2.

**Table 2:** Before-and-after comparisons of energy and nutrient intake.

Subgroup	Variable	n_All n of Pairs with Zero Differences	Pre-Intervention Median [IQR]	Post-Intervention Median [IQR]	Diff Post- vs. Pre-Intervention [IQR]	*p*	RBC
Total	Energy	19	1592.0	1522	−160.0	*p* = 0.286	−0.284
		0	[727.0]	[480.5]	[499.0]		
Total	Carbohydrate	19	216.4	210.5	−22.4	*p* = 0.153	−0.379
		0	[91.3]	[76.6]	[53.5]		
Total	Fat	19	56	60.2	−4.2	*p* = 0.344	−0.253
		0	[21.6]	[17.2]	[20.6]		
Total	Protein	18	58.8	61.7	−3	*p* = 0.459	−0.205
		−1	[29.7]	[36.5]	[15.0]		
Total	Total Fiber	19	11	10.7	−0.4	*p* = 0.387	−0.232
		0	[9.6]	[7.6]	[3.6]		
Female	Energy	15	1896.0	1829	−218.0	*p* = 0.182	−0.4
(Sex = 1)		0	[711.0]	[598.5]	[329.5]		
Female	Carbohydrate	15	252.4	217.2	−22.5	*p* = 0.050	−0.583
(Sex = 1)		0	[93.4]	[74.6]	[64.8]		
Female	Fat	15	66.3	62.9	−6.9	*p* = 0.182	−0.4
(Sex = 1)		0	[24.6]	[15.8]	[17.0]		
Female	Protein	15	66.1	74.5	−3	*p* = 0.670	−0.133
(Sex = 1)		0	[26.9]	[30.5]	[16.6]		
Female	Total Fiber	15	15.8	13.8	−1.7	*p* = 0.222	−0.367
(Sex = 1)		0	[8.0]	[7.4]	[3.8]		

Pre- and postintervention comparisons were performed via the paired Wilcoxon signed-rank test. Given the small sample size and potential violation of normality assumptions, we selected a nonparametric approach. Effect sizes were quantified via rank–biserial correlation (RBC). The number of pairs with zero differences (ties) was recorded, and the effective sample size (n_eff_nonzero) was reported.

**Table 3:** Comparison of nutrient intake before and after energy adjustment (per 1000 calories).

Subgroup	Variable	n_All n of Pairs with Zero Differences	Pre-Intervention Median [IQR]	Post-Intervention Median [IQR]	Diff Post- vs. Pre-Intervention [IQR]	*p*	RBC
Total	Carbohydrate	19	130	127.1	0.453	*p* = 0.763	−0.084
		(0)	[15.2]	[12.7]	[19.4]		
Total	Fat	19	34.3	36.2	−0.132	*p* = 0.763	0.084
		(0)	[7.0]	[5.5]	[8.4]		
Total	Protein	19	35.8	37.2	0.023	*p* = 0.324	0.263
		(0)	[5.0]	[7.5]	[7.3]		
Total	Fiber	19	7.6	7.2	−0.150	*p* = 0.984	−0.011
		(0)	[2.7]	[2.8]	[1.2]		
Female	Carbohydrate	15	130	127.1	2	*p* = 0.712	−0.117
(Sex = 1)		(0)	[17.1]	[12.5]	[21.6]		
Female	Fat	15	34.4	36	−0.859	*p* = 0.887	0.05
(Sex = 1)		(0)	[6.4]	[5.5]	[9.1]		
Female	Protein	15	35.8	39.5	3	*p* = 0.065	0.55
(Sex = 1)		(0)	[4.1]	[5.2]	[6.6]		
Female	Fiber	15	7.9	7.5	−0.051	*p* = 0.755	0.1
(Sex = 1)		(0)	[2.9]	[2.9]	[1.3]		

Pre- and postintervention comparisons were performed via the paired Wilcoxon signed-rank test. Given the small sample size and potential violation of normality assumptions, we selected a nonparametric approach. Effect sizes were quantified via rank–biserial correlation (RBC). The number of pairs with zero differences (ties) was recorded, and the effective sample size (n_eff_nonzero) was reported.

**Table 4:** Before-and-after comparison of physical characteristics (Body fat percentage, BMI, BW, and SMI).

Subgroup	Variable	n_All n of Pairs with Zero Differences	Pre-Intervention Median [IQR]	Post-Intervention Median [IQR]	Diff Post- vs. Pre-Intervention [IQR]	*p*	RBC
Total	BMI	14	24.3	24.6	0	*p* = 0.176	0.419
		(5)	[6.9]	[6.9]	[0.450]		
Total	BW	16	59.9	61	0.1	*p* = 0.155	0.412
		(3)	[23.5]	[22.8]	[1.1]		
Total	SMI	19	6.2	6.3	−0.008	*p* = 0.305	−0.274
		(0)	[1.3]	[1.5]	[0.201]		
Total	BF	19	16.7	32.8	13	*p* < 0.001	0.979
		(0)	[13.7]	[12.4]	[4.9]		
Female	BMI	11	21.8	21.6	0	*p* = 0.721	0.136
(Sex = 1)		(4)	[6.1]	[6.1]	[0.350]		
Female	BW	12	51.8	51.3	0	*p* = 0.724	0.128
(Sex = 1)		(3)	[20.3]	[20.4]	[0.950]		
Female	SMI	15	6	6.1	−0.008	*p* = 0.268	−0.333
(Sex = 1)		(0)	[0.700]	[0.708]	[0.257]		
Female	BF	15	15.5	33.2	13.1	*p* < 0.001	0.983
(Sex = 1)		(0)	[14.1]	[14.1]	[3.9]		

Pre- and postintervention comparisons were performed via the paired Wilcoxon signed-rank test. Given the small sample size and potential violation of normality assumptions, we selected a nonparametric approach. Effect sizes were quantified via rank–biserial correlation (RBC). The number of pairs with zero differences (ties) was recorded, and the effective sample size (n_eff_nonzero) was reported.

**Table 5:** Before-and-after comparison of HbA1c and lipid levels.

Subgroup	Variable	n_All n of Pairs with Zero Differences	Pre-Intervention Median [IQR]	Post-Intervention Median [IQR]	Diff Post- vs. Pre-Intervention [IQR]	*p*	RBC
Total	HbA1c	19	6.9	6.7	−0.200	*p* = 0.009	−0.689
		(0)	[0.950]	[0.800]	[0.500]		
Total	LDLC	19	106	110	4	*p* = 0.481	0.189
		(0)	[33.0]	[34.0]	[13.5]		
Total	TG	18	134	120	−9	*p* = 0.239	−0.322
		(1)	[55.5]	[42.0]	[37.0]		
Total	HDLC	18	55	53	−1	*p* = 0.540	−0.17
		(1)	[18.5]	[17.5]	[6.0]		
Female	HbA1c	15	6.9	6.7	−0.200	*p* = 0.014	−0.725
(Sex = 1)		(0)	[0.850]	[0.800]	[0.400]		
Female	LDLC	15	106	115	3	*p* = 0.629	0.15
(Sex = 1)		(0)	[33.0]	[41.5]	[15.5]		
Female	TG	14	134	121	−1	*p* = 0.593	−0.171
(Sex = 1)		(1)	[59.5]	[40.5]	[34.0]		
Female	HDLC	15	57	55	−2	*p* = 0.550	−0.183
(Sex = 1)		(0)	[22.5]	[23.0]	[8.0]		

Pre- and postintervention comparisons were performed via the paired Wilcoxon signed-rank test. Given the small sample size and potential violation of normality assumptions, we selected a nonparametric approach. Effect sizes were quantified via rank–biserial correlation (RBC). The number of pairs with zero differences (ties) was recorded, and the effective sample size (n_eff_nonzero) was reported.

## Data Availability

The datasets presented in this article are not readily available because the data are part of an ongoing study.
